# Seeing clearly: why gender and sexual diversity must remain in eating disorder science

**DOI:** 10.1007/s40519-025-01772-x

**Published:** 2025-12-11

**Authors:** Paolo Meneguzzo, Ludovica Ragozino, Emilia Manzato, Lorenzo M. Donini, Patrizia Todisco

**Affiliations:** 1 Department of Neuroscience, University of Padova, Padua, Italy; 2 Padova Neuroscience Center, University of Padova, Padua, Italy; 3 U.O.S.D. Eating Disorder Unit, Mental Health Department, ASL Napoli 2 Nord, Naples, Italy; 4 Eating Disorders Center, Private Hospital Salus, Ferrara, Italy; 5 Experimental Medicine Department, Food Science and Human Nutrition Research Unit, Sapienza University of Rome, Viale Regina Elena, 324, 00161 Rome, Italy; 6 Psycho-nutritional Center, Vicenza, Italy; 7 Psycho-nutritional Center, Verona, Italy

For decades, the archetype of the eating disorder patient has been captured in a single, enduring image: the “SWAG” stereotype—short for skinny, white, affluent girl [1]. This figure has dominated clinical descriptions, media portrayals, and research samples alike. But this stereotype is not just a shorthand—it is a constraint. Originally coined to critique the homogeneity of eating disorder representation, the SWAG construct has contributed to a pervasive bias in both clinical and research settings, influencing who is screened, diagnosed, and referred for treatment [2, 3]. As a result, individuals who fall outside this archetype—such as people of color, men, lower-income individuals, and sexual and gender minorities—have historically been overlooked, misdiagnosed, or under-treated, despite comparable or even elevated levels of eating disorder risk [4]. While early research may have benefited from identifying common patterns, continued reliance on this narrow lens has produced a scientific and clinical myopia, obscuring the full diversity of bodies, identities, and experiences affected by disordered eating.

The consequences have been significant. This constrained lens has distorted risk assessment, led to the underdiagnosis of individuals who fall outside dominant stereotypes, and reinforced stigma that silences those most in need of support. Experimental studies have demonstrated that clinicians are significantly less likely to identify disordered eating behaviors as indicative of an eating disorder when the individual is presented as a person of color—particularly African American—even when the symptom profile is identical to that of a white patient [5]. These findings underscore how implicit biases rooted in narrow stereotypes can directly shape clinical decision-making and perpetuate disparities in detection, diagnosis, and care. Thus, it is time to expand the frame and acknowledge the vital role of gender identity and sexual orientation in our understanding of eating disorders [6]. These variables are not peripheral—they are central to patients’ lives and to the evolution of our science.

Recent years have seen international pushback against the inclusion of gender and sexual diversity in health research and care, particularly in fields like reproductive health, HIV, and mental health. These movements—often fueled by anti-gender rhetoric and political campaigns—have influenced institutional policies and public discourse, threatening the visibility and protection of Lesbian, Gay, Bisexual, Transgender, Queer, Intersex, Asexual (LGBTQIA+) populations in several regions [7]. Though often originating in specific geopolitical contexts, these movements can have far-reaching impacts, including in Europe. This is not about importing controversies—it is about affirming the fundamental right of all people to be seen, studied, and cared for [8].

Eating disorders are not gender-neutral, nor are they evenly distributed. Research consistently shows elevated risk among sexual and gender minorities [9, 10]. Transgender adolescents are significantly more likely than their cisgender peers to receive an eating disorder diagnosis, according to a recent meta-analysis [11]. This is in line with broader survey evidence indicating a high prevalence of disordered eating behaviors among transgender college students [12]. Gay and bisexual boys and men report higher body dissatisfaction and greater use of compensatory behaviors than heterosexual peers, while lesbian and bisexual girls and women demonstrate complex patterns of both risk and resilience—findings observed across both adolescent and adult populations [13, 14]. Yet sexual and gender minorities remain vastly underrepresented in research.

This exclusion is not just statistical—it is clinical. Queer individuals often develop disordered eating in response to unique social stressors: minority stress, internalized stigma, body dysphoria, victimization, and lack of access to affirming care [11]. Although research is still limited, existing studies suggest that intersex and asexual individuals may experience distinct challenges related to body image, identity, and mental health, which are likely to influence eating behaviors [15, 16]. For some transgender people, restrictive eating may become a way to suppress secondary sex characteristics or delay puberty in the absence of medical transition (e.g., through puberty blockers or hormones) [17]. In other cases, eating disorder behaviors may emerge or intensify in response to BMI-based eligibility criteria for gender-affirming care, which can create additional pressure to modify one’s body through non-medicalized means [18]. These mechanisms demand recognition, ignoring them risks rendering standard treatment settings ineffective for those who most need support [19].

Language plays a crucial role. When research omits questions on gender identity or sexual orientation—or avoids these terms in favor of so-called “neutrality”—it sacrifices accuracy for the illusion of objectivity [20]. These omissions reflect a hierarchy of visibility, reinforcing the idea that some patients count more than others. And they carry consequences in how we diagnose, treat, and support recovery. Science is never neutral—it reflects the assumptions and variables it chooses to prioritize. As such, the exclusion of gender and sexual diversity from research is not a passive omission but an epistemological distortion. The exclusion of gender and sexual diversity from research is not just a gap; it shapes the production of knowledge in ways that can obscure or distort reality [2, 21]. The lack of affirming, inclusive frameworks contributes to poorer mental health outcomes and diminished care engagement among transgender individuals—making the stakes of exclusion unmistakably clinical.

The consequences of this exclusion are tangible. Group therapy environments that do not recognize or accommodate gender diversity may inadvertently re-traumatize participants, particularly those whose identities have been historically invalidated or pathologized [22, 23]. Similarly, psychoeducational materials that rely exclusively on cisnormative and heteronormative narratives risk alienating patients whose identities fall outside these norms, thereby undermining the therapeutic alliance, eroding trust, and reducing engagement in treatment [24]. These are not hypothetical concerns—they are daily challenges encountered by gender- and sexually diverse individuals navigating systems of care that were not built with them in mind. To move forward, we must shift from inclusion as an afterthought to inclusion as a foundational principle of scientific inquiry and clinical design.

Italy and Europe are not immune to broader anti-gender narratives [25]. These movements—often framed as efforts to protect children or defend “biological truth”—seek to conflate sex and gender, promote essentialist views of identity, and delegitimize the experiences of transgender, intersex, and non-heterosexual individuals [26]. Their influence extends beyond politics, shaping public discourse, institutional agendas, and even scientific inquiry [27–29]. In research environments, this pressure can foster self-censorship or avoidance, reinforcing normative assumptions. Silence, in this context, becomes a form of complicity. In the field of eating disorders—where identity, embodiment, and sociocultural ideals intersect—such erasures risk narrowing our scientific lens and undermining inclusive, effective care. As members of the LGBTQIA+ Special Interest Group within the Italian Society for the Study of Eating Disorders (SISDCA), we are committed to resisting this erasure. This is not about ideology—it is about clinical excellence, scientific integrity, and human dignity. Our mission is to improve outcomes for all individuals affected by eating disorders. That mission cannot be fulfilled through exclusionary frameworks.

Research is not just an academic pursuit—it is a map of reality. Clinical care follows that map. When LGBTQIA+ experiences are left uncharted, care gaps emerge. Patients do not see themselves reflected in treatment models, and clinicians are left unprepared. The result is a healthcare system where too many people fall through the cracks. As documented in recent qualitative research, transgender and nonbinary individuals with eating disorders often experience treatment settings as misaligned with their needs, particularly due to a lack of trans-affirming practices, assumptions rooted in binary gender norms, and the erasure of their lived experiences [30]. These systemic gaps not only compromise therapeutic engagement, but also reinforce feelings of marginalization and mistrust [31]. Without research that actively centers these perspectives, clinical frameworks remain incomplete—and in some cases, iatrogenic.

The European research community has a proud tradition of advancing equity and inclusion. We must now protect that legacy. Journals, institutions, funders, and professional societies all share responsibility. This means defending the right to study gender and sexuality as legitimate scientific variables. It means publishing research on underrepresented groups. And it means training future clinicians and scholars to approach diversity not as a challenge, but as a foundation.

We are at a pivotal moment—one in which the epistemological choices we make will determine not only the future of eating disorder research, but also who is seen, who is served, and who is left behind. The health sciences have long been shaped by binary models and normative assumptions that flatten complexity and marginalize those who live outside presumed categories. Rising to meet the richness of lived experience requires more than inclusive rhetoric—it calls for methodological transformation, epistemic humility, and relational accountability. This includes adopting multidimensional frameworks for measuring sex, gender identity, and sexual orientation [32], and integrating community-driven guidelines that center the expertise of transgender, intersex, and gender-diverse researchers [33]. It also means reforming institutional infrastructures—such as electronic health records—that currently render queer and trans lives invisible [34]. Inclusion is not merely an ethical stance; it is a scientific necessity. Precision, rigor, and equity are not mutually exclusive—they are interdependent. If our maps of reality are to guide just and effective care, they must be drawn with the full range of human variation in mind. To those who claim that acknowledging gender and sexual diversity is a political act, we say this: so is exclusion. The difference is that inclusion expands both knowledge and care—exclusion restricts them. As feminist scholars have long argued, scientific inquiry is never neutral; it is shaped by the cultural, social, and institutional forces in which it is embedded. Feminist epistemologies invite us to interrogate whose bodies and experiences are rendered visible, whose knowledge is considered legitimate, and whose suffering is deemed treatable [35]. In the context of eating disorder research, refusing to engage with gender and sexual diversity is not a neutral omission—it is a political choice with clinical consequences.

As we close, we return to the image that opened this editorial. For years, the field has been dominated by the silhouette of the SWAG figure—thin, white, cisgender, and privileged. But the true landscape of eating disorders is not a silhouette. It is a stained-glass window: multifaceted, colorful, and alive with intersecting shapes and shifting light. To focus on a single pane is to miss the full depth and truth of the picture—the richness of human diversity and the varied realities of those affected by eating disorders. This means embracing an intersectional approach—one that recognizes how systems of oppression such as racism, classism, ableism, and cisheteronormativity intersect and compound vulnerability [36]. Identities are not simply layered but interdependent, with intersecting systems of power shaping how individuals experience illness, recognition, and recovery. As recent scholarship has emphasized, intersectional approaches are essential in eating disorder research to understand how gender, sexuality, race, and other axes of identity converge to produce unique risks and barriers to care [14, 35]. Without this lens, our models remain partial—and our interventions incomplete. In research, as in care, visibility is healing. It affirms the full humanity of every person who walks through the door of a clinic or contributes to the pages of a dataset. To erase identity is to erase need. Today, we choose to see. We choose to include. We choose to listen—to all bodies, all identities, and all lives. But listening must lead to action. The path forward calls us to redesign our studies to meaningfully capture gender identities and sexual orientations, adapt treatment models to be inclusive and affirming, and train clinicians to meet the needs of those too often overlooked. The future of eating disorder science depends on what—and whom—we are willing to include.

## Data Availability

No datasets were generated or analyzed during the current study.
